# Effectiveness of the Bidimensional Bracket System in Miniscrew-Supported en Masse Retraction of the Maxillary Anterior Teeth: A Preliminary Clinical Report

**DOI:** 10.7759/cureus.109738

**Published:** 2026-05-27

**Authors:** Abdulmalek M.H. Almasri, Mohammad Y. Hajeer, Ahmad S. Burhan

**Affiliations:** 1 Department of Orthodontics, Faculty of Dentistry, Damascus University, Damascus, SYR

**Keywords:** bidimensional bracket system, class ii malocclusion, en masse retraction, orthodontic camouflage, orthodontic mini-screw, torque control

## Abstract

Introduction

In adult Class II Division 1 cases treated by orthodontic camouflage, the extraction of the maxillary first premolars is commonly followed by retraction of the anterior segment. Achieving this movement efficiently while maintaining adequate torque control remains a major clinical concern.

Materials and methods

This preliminary clinical report evaluated the performance of a bidimensional bracket system combined with miniscrew-supported anchorage in seven adult patients treated with the extraction of the maxillary first premolars followed by en masse retraction of the upper anterior segment. After completion of leveling and alignment, retraction was initiated using nickel-titanium closed-coil springs delivering 250 g of force per side. The assessed outcomes were the change in maxillary incisor torque on cephalometric images, measured using the bracket-archwire angle, and the rate of anterior retraction on 3D digital models, measured as the distance from the incisal edge of the maxillary central incisors to the medial endpoint of the third right palatal rugae. The statistical analysis was exploratory, and 95% CIs were calculated for the main clinical estimates.

Results

Repeated measurements demonstrated high reliability, with intraclass correlation coefficients ranging from 0.971 to 1 and no statistically significant systematic error. The mean change in maxillary incisor torque following retraction was 1.54 ± 0.27° (95% CI: 1.29-1.79°). The monthly retraction rate ranged from 0.67 ± 0.05 to 0.75 ± 0.08 mm/month, with an overall mean of 0.71 ± 0.04 mm/month (95% CI: 0.67-0.75). Total retraction reached 5.60 ± 0.72 mm, corresponding to 88.32% of the extraction space, and the mean duration required to complete en masse retraction was 7.89 ± 1.11 months. No patient withdrawal, miniscrew failure, peri-miniscrew inflammation requiring screw removal, or appliance-related complication interrupting treatment was recorded.

Conclusions

Within the limitations of this exploratory preliminary report, the bidimensional bracket system used with miniscrew-supported anchorage appeared to preserve torque well, along with a controlled and clinically acceptable rate of anterior retraction. This approach may represent a useful biomechanical option for improving 3D control during space closure.

## Introduction

In adult Class II cases treated with orthodontic camouflage, maxillary first premolar extraction is commonly selected when anterior retraction and sagittal dental correction are required [1]. After space creation, the anterior teeth may be retracted either together in an en masse approach supported by reinforced anchorage or sequentially after separate canine retraction [2]. En masse retraction may reduce some of the unwanted canine movements associated with a staged approach, such as tipping and rotation, and may therefore simplify the subsequent finishing of the anterior segment [3,4]. During retraction, preserving maxillary incisor torque is essential for maintaining anterior guidance, overjet, overbite, and the final sagittal relationship of the dentition [5].

Torque control in conventional bracket systems has been widely investigated, as anterior retraction across extraction spaces may result in loss of maxillary incisor torque and affect the quality of the final occlusal outcome [6]. This loss is mainly due to the force application point being occlusal to the center of resistance and to the inherent play between the bracket slot and the archwire, which limits full torque expression [5]. Additionally, most torque control methods tend to increase chair time, may lead to patient discomfort, and often produce unpredictable outcomes [7]. Sliding mechanics can also cause side effects such as tooth tipping, torque loss, poor vertical control, anchorage loss, and incisor extrusion [8]. To address these issues, the bidimensional system was created; it uses two types of brackets with different slot sizes to keep precise 3D control over the front teeth while making it easier to move the back teeth [9]. Despite the mechanical advantages of conventional bracket systems in controlling tooth movement in three dimensions, torque loss during en masse retraction remains a significant clinical challenge [10].

Several modifications have been proposed to enhance torque control, such as using rectangular archwires, incorporating compensatory bends, or employing miniscrews for additional anchorage; however, these approaches are often technically complex, increase chairside time, and may result in greater patient discomfort [11]. In this context, the bidimensional bracket system represents a promising approach by employing smaller slots on anterior teeth to maximize torque expression and larger slots on posterior teeth to reduce friction during sliding mechanics [12]. This design aims to improve anterior torque control while simultaneously facilitating efficient posterior tooth movement, thereby minimizing undesirable effects such as tipping, extrusion, or anchorage loss [12]. Although the bidimensional system has been described for several decades, clinical evidence regarding its effectiveness remains limited, particularly in the context of miniscrew-supported en masse retraction. Given the increasing reliance on temporary anchorage devices (TADs) to reinforce anchorage and enhance treatment efficiency [13], several adjunctive acceleration methods have also been investigated to shorten the duration of en masse retraction of the upper anterior teeth, although their reported effectiveness remains variable across studies [14]. In this context, it is essential to assess whether the bidimensional approach offers a significant advantage in torque control, treatment efficiency, and clinical side effects.

Therefore, the present preliminary pilot study was designed to assess the clinical performance of the bidimensional bracket system during miniscrew-supported en masse retraction of the upper anterior teeth, with particular emphasis on torque control and the rate of tooth movement.

## Materials and methods

Study design and setting

This pilot interventional clinical study aimed to assess the clinical performance of a bidimensional bracket system in the en masse retraction of the upper anterior teeth. The study was carried out at the Department of Orthodontics, Faculty of Dentistry, Damascus University, Syria. This pilot study was ethically approved as part of a larger clinical study protocol investigating the effectiveness of a bidimensional bracket system, which was approved by the Research Ethics Committee of the Faculty of Dentistry, Damascus University (DN-300625-479). Given the exploratory pilot nature of this study, which aimed to provide preliminary clinical data on the performance of the bidimensional system and to generate baseline outcome estimates for future controlled studies, the trial protocol was not registered, and no concurrent control group was included.

Patient recruitment

Seven adult patients with Angle Class II Division 1 malocclusion were recruited from the orthodontic patient records at the Faculty of Dentistry, Damascus University. Patients were eligible for inclusion if they were 18-28 years old, presented with a skeletal Class II relationship defined by an ANB angle of 5-8°, had dental Class II Division 1 malocclusion according to Angle’s classification, and required extraction of the maxillary first premolars as part of the treatment plan. Patients were not considered for inclusion if they presented with an overjet exceeding 8 mm, an anterior deep bite, moderate or severe crowding, an absence of upper permanent teeth, a history of previous orthodontic treatment, systemic diseases or ongoing medication use, poor oral hygiene, or periodontal pathology. Before participation, all patients received a full explanation of the study protocol. After the study procedures had been explained, all participants signed a written informed consent form.

Treatment sequence

Extraction of First Premolars

The maxillary first premolars were extracted before bonding the fixed orthodontic appliance, in accordance with the planned treatment protocol.

Anchorage Reinforcement

Anchorage was reinforced using bilateral self-drilling orthodontic miniscrews measuring 1.6 × 8 mm (3S screw, HUBIT Orthodontics, Gyeonggi, South Korea). The screws were placed between the roots of the maxillary second premolars and first molars on both sides.

Leveling and Alignment

The study employed Pinnacle™ McLaughlin, Bennett, and Trevisi (MBT) prescription brackets with pre-adjusted fixed orthodontic appliances (Pinnacle™, Ortho Technology, West Columbia, SC, USA). MBT prescription brackets were used in two slot dimensions: 0.018-inch brackets were placed on the anterior teeth, while 0.022-inch brackets were placed on the posterior teeth. During this phase, archwires from Ortho Technology were replaced at three-week intervals, progressing through the following sequence: 0.014-inch NiTi, 0.016 × 0.022-inch NiTi, 0.017 × 0.025-inch NiTi, 0.018 × 0.025-inch NiTi, and finally 0.018 × 0.025-inch stainless steel. Three weeks after placement of the final stainless-steel archwire, crimpable hooks were attached distal to the maxillary lateral incisors.

En Masse Retraction of the Upper Anterior Teeth

**Figure 1:**
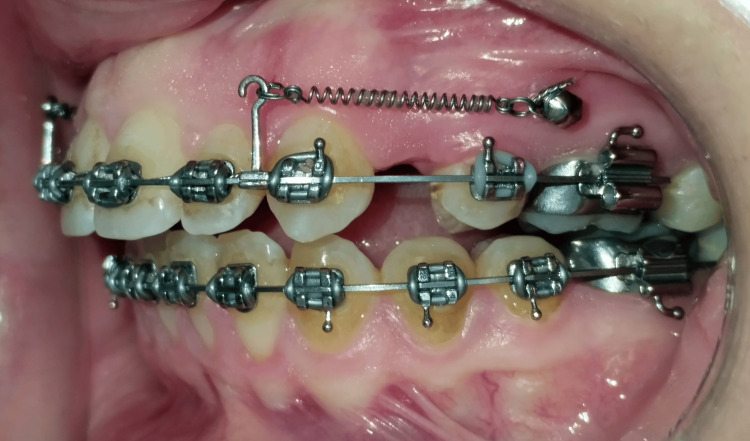
Lateral intraoral view recorded at the beginning of en masse retraction

Following the leveling and alignment phase, retraction of the upper anterior segment was performed using NiTi closed-coil springs (NT3® closed coil, American Orthodontics, Sheboygan, WI, USA), stretched bilaterally between the crimpable hooks and the miniscrews (Figure 1). A force of 250 g was applied to each side and verified every three weeks with an intraoral force gauge (040-711-00; Dentaurum GmbH & Co. KG, Ispringen, Germany).

Outcome measures

Primary Outcome Measure: Change in Upper Incisors Torque Following Retraction

**Figure 2:**
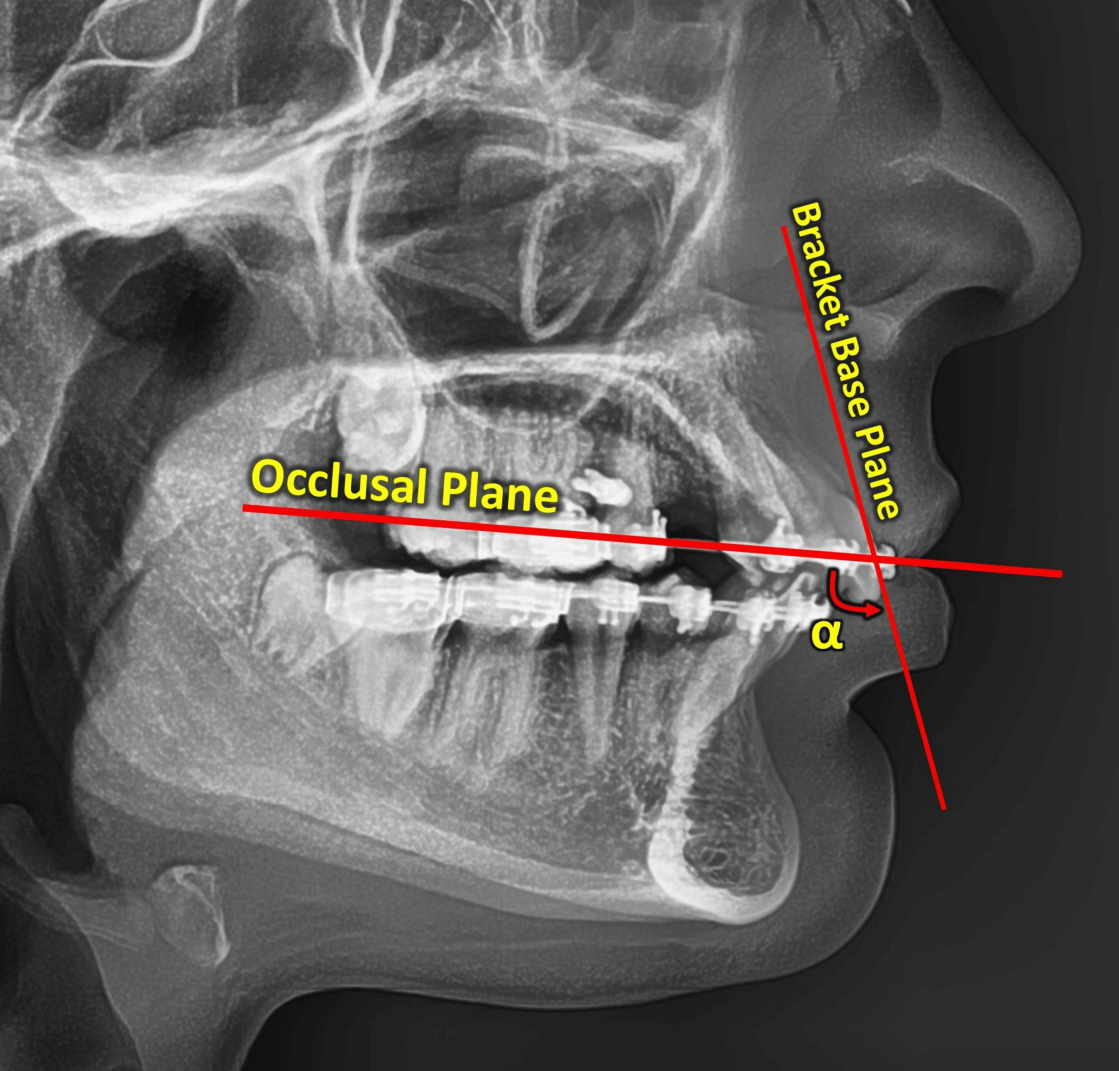
Lateral cephalometric image illustrating the measurement of the bracket-archwire angle (α) used to assess torque expression

The amount of change in the value of the maxillary incisor torque after the completion of the en masse retraction was measured on cephalometric images. Two images were taken: the first at T0, after leveling and alignment, and the second at T1, after en masse retraction. The change in torque was quantified using ImageJ software (National Institutes of Health, Bethesda, MD, USA), following the analytical procedure described by Li et al. [12]. Briefly, the angle formed between the base of the maxillary central incisor bracket and the working rectangular archwire was measured. This angle (α) directly represents the interaction between the archwire and the bracket slot (Figure 2). The torque value of the maxillary central incisor (TQ_U1) was then calculated as α minus 90°. This measurement approach was selected to minimize the influence of tooth morphology and bracket positioning and to provide a direct assessment of effective torque expression at the bracket-wire interface.

Secondary Outcome Measures: Rate and Duration of the en Masse Retraction

**Figure 3:**
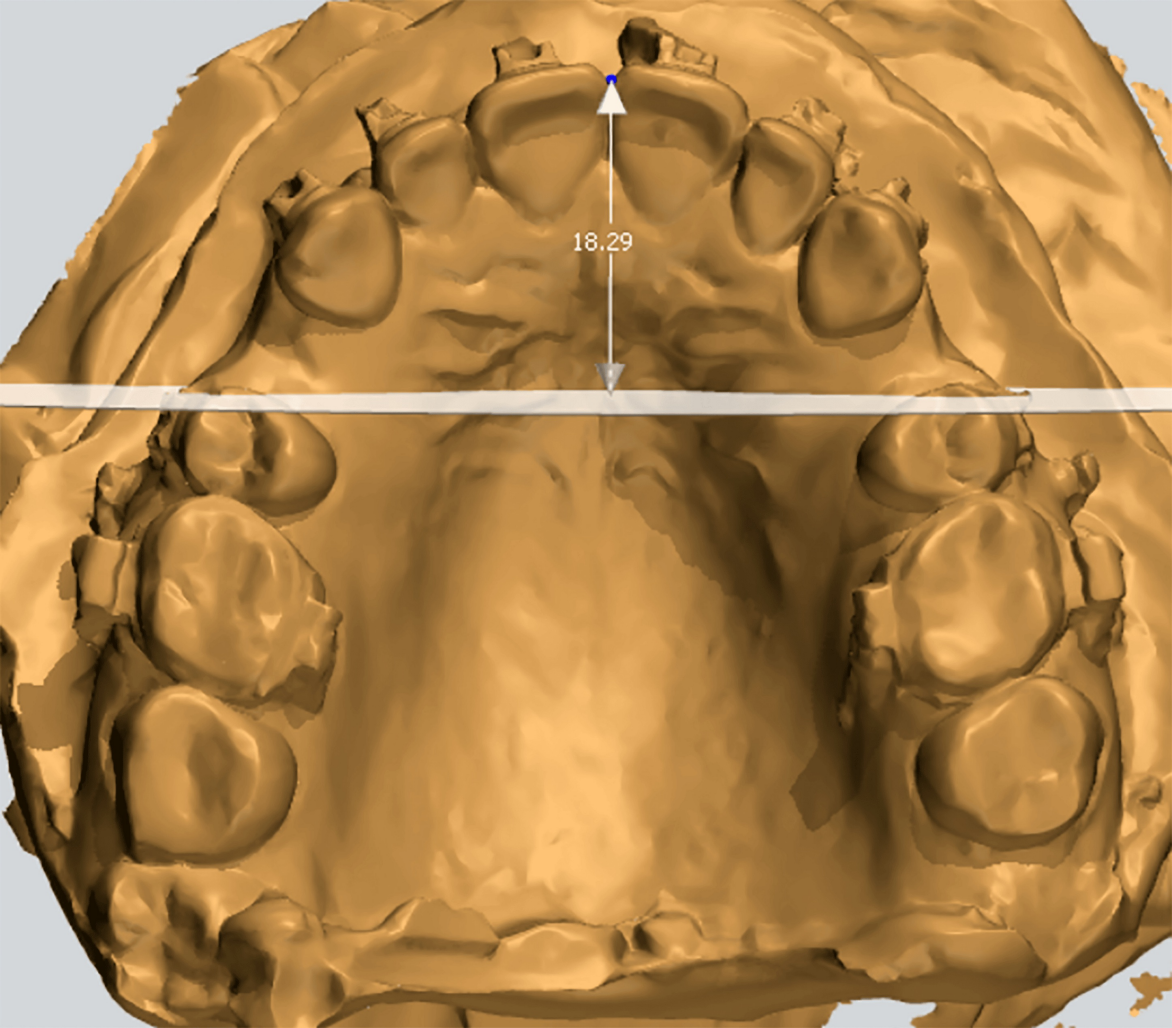
3D digital model in 3Shape Ortho Analyzer software showing the linear measurement used to quantify incisor retraction

Study models were used to assess the monthly amount of upper anterior tooth retraction. For each interval, the rate of retraction was calculated as the retraction distance in millimeters divided by the elapsed time in months. Model casts derived from alginate impressions were obtained at predetermined intervals: T0, at completion of the leveling and alignment phase; T1, one month following initiation of en masse retraction; T2, after two months; T3, after three months; T4, after four months; T5, after five months of en masse retraction; and TF, at the conclusion of the retraction phase, defined by attainment of a Class I canine relationship and an appropriate overjet. All dental casts were digitally scanned to generate 3D models, and the measurements were performed digitally using 3Shape Ortho Analyzer software (3Shape, Copenhagen, Denmark). The distance from the incisal edge of the maxillary central incisors to the medial endpoint of the third right palatal rugae was measured on 3D digital models to quantify the extent of incisor retraction (Figure 3). The monthly retraction rate was then calculated by dividing the retraction distance by the elapsed time.

Measurement reproducibility and reliability assessment

To improve measurement reproducibility, all lateral cephalometric images and digital dental models were evaluated according to predefined measurement protocols. For torque assessment, radiographs were obtained according to the standardized cephalometric positioning protocol routinely used at the Department of Orthodontics. The visibility of the maxillary central incisor bracket, the rectangular working archwire, and the bracket-archwire interface was verified before performing the measurements. The two reference elements used for torque assessment were the bracket base plane and the long axis of the working rectangular archwire.

Before formal data collection, the examiner was trained on the measurement procedures using records not included in the final analysis. All measurements were performed by the same examiner under similar conditions using ImageJ software for cephalometric measurements and 3Shape Ortho Analyzer software for digital model measurements. To assess intra-examiner reproducibility, all cephalometric and dental model measurements were repeated 10 days after the first evaluation, with the examiner blinded to the initial measurement values. Intraclass correlation coefficients (ICCs) were used to assess random error, while paired-sample t-tests were applied to identify systematic error.

Statistical analysis

Given the preliminary nature of this uncontrolled clinical report and the small number of included patients, the statistical analysis was considered exploratory and hypothesis-generating. No confirmatory between-group hypothesis testing was planned. Continuous variables were summarized using means, standard deviations, medians, minimum, and maximum values. For the main clinical outcomes, two-sided 95% CIs were calculated using the t distribution. The primary clinical estimate was the mean change in maxillary incisor torque after en masse retraction, while secondary estimates included the monthly retraction rate, total amount of anterior retraction, and duration of retraction. Because no concurrent control group was included, comparative between-group effect size estimates were not applicable. Measurement reproducibility was assessed using ICCs, and paired-sample t-tests were used to detect systematic differences between repeated measurements. Statistical significance was set at p < 0.05.

## Results

Baseline sample characteristics

Following informed consent, seven patients (four females and three males) with a mean age of 20.77 ± 0.93 years were recruited for this pilot study. Upon completion of the follow-up period, the data obtained from these participants were analyzed. Table 1 summarizes the patients’ baseline characteristics. All seven patients completed the planned en masse retraction phase and were included in the final analysis. No patient withdrew from the study. No miniscrew failure, peri-miniscrew inflammation requiring screw removal, or appliance-related complication that interrupted treatment was recorded. Any transient soft-tissue irritation, when present, was managed with routine oral hygiene reinforcement and did not require miniscrew removal or discontinuation of treatment.

**Table 1:** Baseline characteristics of the sample at the beginning of the treatment

Variable	Value
Number of patients	7
Gender (male/female)	3/4
Age (years) ± SD	20.77 ± 0.93
Pre-retraction extraction space	6.34 ± 0.41

Reliability assessment of the measurement method

The calculated ICCs indicated high reliability for the measurements, with values ranging from 0.971 to 1. Conversely, paired-sample t-tests revealed no statistically significant differences between the two sets of measurements (p > 0.05), indicating negligible, clinically insignificant systematic error.

Change in the upper incisors’ torque following retraction

The change in maxillary incisor torque following en masse retraction was minimal and relatively consistent across the patients studied. The mean torque change was 1.54° ± 0.27°, with a median of 1.53°, and the observed values ranged from 1.33° to 1.72°. The exploratory 95% CI for the mean torque change was 1.29° to 1.79°. This narrow distribution suggests effective torque control during the retraction phase when the bidimensional bracket system was used in combination with miniscrew-supported anchorage.

Rate and duration of the en masse retraction

The descriptive statistics of the en masse retraction rate of the upper anterior teeth are presented in Table 2. The mean monthly retraction rate ranged from 0.67 ± 0.05 mm during the first month (T0-T1) to 0.75 ± 0.08 mm during the fourth month (T3-T4). A relatively consistent retraction rate was observed throughout the retraction period, with mean values of 0.71 ± 0.06 mm/month (T1-T2), 0.69 ± 0.06 mm/month (T2-T3), and 0.73 ± 0.07 mm/month (T4-T5). Overall, the total en masse retraction rate from T0 to TF was 0.71 ± 0.04 mm/month, indicating efficient and stable anterior tooth movement. The limited variation in retraction rates across different time intervals suggests controlled space closure and effective anchorage during miniscrew-supported en masse retraction using the bidimensional bracket system. At the final assessment, total anterior retraction averaged 5.60 ± 0.72 mm, corresponding to nearly 88.32% of the maxillary first premolar extraction space. En masse retraction was completed over a mean period of 7.89 ± 1.11 months. The exploratory 95% CIs were 0.67-0.75 mm/month for the overall retraction rate, 4.93-6.27 mm for total anterior retraction, and 6.86-8.92 months for the duration of en masse retraction.

**Table 2:** Descriptive statistics of the en masse retraction rate (mm/month)

Variable	Intervals	Mean ± SD	Median	Max	Min
Upper anterior teeth retraction rate	T0-T1	0.67 ± 0.05	0.67	0.74	0.60
	T1-T2	0.71 ± 0.06	0.72	0.79	0.62
	T2-T3	0.69 ± 0.06	0.7	0.77	0.60
	T3-T4	0.75 ± 0.08	0.75	0.85	0.64
	T4-T5	0.73 ± 0.07	0.74	0.82	0.62
	T0-TF	0.71 ± 0.04	0.71	0.76	0.66

## Discussion

This preliminary study examined how the bidimensional bracket system performed clinically during miniscrew-supported en masse retraction of the maxillary anterior segment, with particular attention to incisor torque control and retraction rate. The findings suggest a minimal mean torque change of 1.54° ± 0.27° in the maxillary incisors following retraction, alongside a consistent mean monthly retraction rate of 0.71 ± 0.04 mm/month. These results suggest that the bidimensional system, when combined with TADs, can provide effective 3D control during space closure, minimizing undesirable side effects such as loss of torque, tipping, and anchorage loss.

The observed change in torque in this study was relatively small and consistent across patients. This finding is clinically significant, as loss of incisor torque during retraction over large extraction spaces remains a common challenge in sliding mechanics [5,6]. The bidimensional system employs smaller slot dimensions (0.018 inch) on the anterior teeth, which are theoretically designed to enhance torque expression by reducing the play between the archwire and bracket slot [12,15]. Our results align with the biomechanical premise of the system and are comparable to the outcomes reported by Li et al., who found that bidimensional techniques offered stronger anterior torque control in extraction cases [12]. The minimal torque change recorded in our study (max 1.72°, min 1.33°) indicates that the system successfully maintained the labiolingual inclination of the anterior segment during en masse movement, which is crucial for achieving ideal overjet, overbite, and final occlusion [5].

Regarding the rate of tooth movement, the mean monthly retraction rate of 0.71 mm/month and the mean duration of 7.89 ± 1.11 months observed in the present study indicate controlled and clinically acceptable space closure within the range reported for conventional, non-accelerated en masse retraction protocols. Previous studies using miniscrew-supported conventional retraction have reported treatment durations of 8.6-9.4 months in studies by Upadhyay et al. [16-18] and 12.9 months in the trial by Al-Sibaie and Hajeer [2]. More recent randomized evidence also showed comparable conventional values, with Mousa et al. reporting a rate of 0.75 ± 0.06 mm/month in the conventional group [19], while Shaadouh et al. reported a mean retraction duration of 8.11 ± 0.78 months in the traditional group [20]. Hatrom et al. likewise reported a monthly space-closure rate of 0.6 mm in their control group [21]. Taken together, these comparisons suggest that the bidimensional bracket system used in the present study provides a retraction rate comparable to that achieved with conventional non-accelerated mechanics while maintaining effective control during space closure. Such comparisons should be interpreted carefully, given the variation among studies in observation periods, force systems, and methods used to assess tooth movement.

In contrast, higher retraction rates have generally been reported in studies using adjunctive acceleration procedures. Khlef et al. observed monthly rates of 1.26 and 1.38 mm with flapless and conventional corticotomy, respectively [22]. In contrast, Shaadouh et al. reported a rate of 0.97 ± 0.06 mm/month after applying low-intensity direct electrical current [23]. In addition, Mousa et al. reported rates of 1.09 ± 0.13 mm/month with flapless piezocision alone and 1.32 ± 0.19 mm/month when piezocision was combined with low-level laser therapy [19], whereas Hatrom et al. observed a monthly space-closure rate of 1.2 mm in the piezocision group [21]. These findings indicate that the bidimensional system is not primarily an acceleration approach. It should also be noted that not all adjunctive approaches have demonstrated a meaningful acceleration benefit. Al-Bozaie et al. found that platelet-rich plasma was ineffective in increasing the rate of tooth movement or altering the type of movement during mini-implant-based en masse retraction [24]. Rather, its clinical value appears to lie in optimizing biomechanics and preserving anterior control during en masse retraction, rather than maximizing the absolute speed of tooth movement. From a clinical perspective, the choice between the bidimensional system and adjunctive acceleration procedures should therefore depend on the primary treatment objective, whether it is to enhance biomechanical control and torque preservation or to shorten the duration of space closure.

The high reliability of our measurement methods (ICCs 0.971-1) strengthens the validity of these findings. Furthermore, the consistent retraction rate throughout the observation period (Table 2) suggests stable anchorage and controlled force application, with the miniscrews effectively preventing unwanted anchorage loss [8]. The total retraction achieved (5.60 ± 0.72 mm, representing 88.32% of the extraction space) confirms the protocol's efficiency in closing the space.

A noteworthy aspect of modern orthodontic research is the evaluation of patient-centered outcomes [25-27]. Although the present preliminary study did not formally assess pain or discomfort levels, the clinical application of the bidimensional system did not introduce any novel or complex procedures for the patient beyond standard fixed appliance therapy. Future research on this system should incorporate patient-reported outcome measures to fully evaluate its acceptability and impact on patient experience during treatment. In addition to treatment efficiency and patient-reported outcomes, future studies should also evaluate root resorption, as recent evidence has highlighted its importance when assessing adjunctive procedures intended to accelerate orthodontic tooth movement [28].

Limitations

Several limitations should be considered when interpreting the present findings. The small number of participants reflects the preliminary design of the study and reduces both statistical power and the extent to which the results can be generalized. Accordingly, the reported estimates and CIs should be interpreted as exploratory values intended to inform future controlled studies rather than as confirmatory evidence of treatment effectiveness. Because the study did not include a parallel control group, the observed outcomes cannot be attributed exclusively to the bidimensional bracket system. In addition, all patients had Class II Division 1 malocclusion and were treated according to a standardized extraction-based protocol, which may limit the applicability of these findings to other malocclusion patterns or treatment mechanics. Finally, the assessment was confined to the active retraction phase, and the post-treatment stability of incisor torque and arch alignment was not evaluated.

## Conclusions

Within the limitations of this preliminary pilot study, the bidimensional bracket system combined with miniscrew-supported anchorage demonstrated favorable clinical performance during en masse retraction of the upper anterior teeth. It allowed good torque preservation and a stable, clinically acceptable rate of retraction. This approach may therefore represent a useful biomechanical option for improving 3D control during space closure. Further randomized controlled trials with larger sample sizes and appropriate control groups are needed to validate these preliminary findings, compare this system with conventional bracket systems and acceleration-assisted protocols, and evaluate root resorption, long-term stability, and patient-reported outcomes.
